# An Adaptive Filtering Approach Based on the Dynamic Variance Model for Reducing MEMS Gyroscope Random Error

**DOI:** 10.3390/s18113943

**Published:** 2018-11-14

**Authors:** Yanshun Zhang, Chuang Peng, Dong Mou, Ming Li, Wei Quan

**Affiliations:** 1 School of Instrumentation and Optoelectronic Engineering, Beihang University, Beijing 100191, China; zhangyanshun@buaa.edu.cn (Y.Z.); liliyalm@buaa.edu.cn (M.L.); quanwei@163.com (W.Q.); 2 Electronic Engineering Research Institute, China Academy of Engineering Physics, Mianyang 621900, China; md1015@126.com

**Keywords:** MEMS gyroscope, dynamic random error, variance model, Kalman Filter

## Abstract

To improve the dynamic random error compensation accuracy of the Micro Electro Mechanical System (MEMS) gyroscope at different angular rates, an adaptive filtering approach based on the dynamic variance model was proposed. In this paper, experimental data were utilized to fit the dynamic variance model which describes the nonlinear mapping relations between the MEMS gyroscope output data variance and the input angular rate. After that, the dynamic variance model was applied to online adjustment of the Kalman Filter measurement noise coefficients. The proposed approach suppressed the interference from the angular rate in the filtering results. Dynamic random errors were better estimated and reduced. Turntable experiment results indicated that the adaptive filtering approach compensated for the MEMS gyroscope dynamic random error effectively both in the constant angular rate condition and the continuous changing angular rate condition, thus achieving adaptive dynamic random error compensation.

## 1. Introduction

The Micro-Electro-Mechanical Systems (MEMS) gyroscope has advantages of affordable, compactness and low power consumption, which is widely used in the fields of inertial measurement and inertial stabilization with considerably good application prospects [1,2,3,4,5]. However, due to its unique processing technology and material micro-scale effects [6,7], a MEMS gyroscope has considerable errors, which need to be compensated [8,9,10,11]. MEMS gyroscope errors contain system errors and random errors. System errors such as bias and scale are compensated by laboratory turntable calibration [12] and specific movement state-based system-level calibration. However, random errors have characteristics of uncertainty and randomness [13], which cannot be fully compensated by analysis methods, yet can be restrained with a filtering method [14,15].

To describe random signals, the time sequence model is an effective approach [16]. According to differences in the random signal statistic characteristics, relevant researchers utilized the Auto Regressive and Moving Average (ARMA) model and the Auto Regressive Integrated Moving Average (ARIMA) model respectively to describe stationary data and non-stationary data [17], and combined them with the Kalman Filter to filter the random data [18]. In previous work, the ARMIA model-based Kalman filtering commonly adopted prior data to calculate the measurement noise coefficients, which has a preferable filtering performance on static gyroscope output data [19,20]. Under a maneuvering condition with different angular rates, however, the statistic feature of gyroscope output data has a relation to gyroscope motion [21,22]. Moreover, it is shown in Section 2, that the statistic characteristics of gyroscope dynamic random errors have certain mapping relations with angular rate. In practical application, carrier angular rates are always in dynamic variation. Therefore, the gyroscope dynamic random errors modeling compensation approach is a key technology that needs to be developed currently.

For the problem of gyroscope dynamic random error, researchers have studied dynamic error characteristic analysis and the filtering method. References [23,24] applied the dynamic Allan variance method to the analysis ring laser gyroscope (RLG) and the MEMS gyroscope’s dynamic random error characteristics. Reference [25] utilized the Fading Kalman Filtering method to restrain the MEMS gyroscope random error.

The author worked on MEMS gyroscope testing for many years, and it was found that statistical characteristics of gyroscope random error had a relation with angular rates [21,22]. Moreover, in Section 2, a certain functional relation between data variance and angular rates was found which could be applied to gyroscope dynamic random error compensation. Therefore in this paper, based on previous research results in reference [17], the ADIS16488 gyroscope is taken as an example. A dynamic variance model showing relations between data variance and angular rate is built. Furthermore, the model was utilized to adjust the measuring noise coefficients of Kalman Filtering (KF) online. Experiments were designed to verify the proposed method in Section 4.

## 2. Angular Rate Related Dynamic Variance Model

The angular related dynamic variance model is a mapping relation between input angular rate and output MEMS Gyroscope Random Signal variance. Reference [24] adopted the dynamic David Allan Variance method to analysis the ADIS16255 MEMS gyroscope output random signal, concluding that the gyroscope output signal variance fluctuates in practical engineering applications. On the basis of this conclusion, the MEMS gyroscope ADIS16488 was chosen as a study object. By generating different angular rates on a turntable, MEMS gyroscope output signals at each rate were collected. Then the dynamic variance model $V_{\omega }=f(\omega )$ between random signal variance $V_{\omega }$ and angular rate ω were studied, which is the foundation of gyroscope random signal filtering. ADIS16488 and experimental turntable parameters are briefly discussed in Section 4.1.

The dynamic variance model was built with four procedures:

### 2.1. Equipment Installation

The ADIS16488 is fixed by a mounting fixture to the interior frame of the three-axis turntable. The interior frame and the middle frame are adjusted to a vertical position to position the gyroscope’s Z sensitive craft point vertically upwards.

### 2.2. Data Acquisition

The turntable is controlled to generate different angular rates. After the rotation rate data show to be smooth and steady, the gyroscope Z axis’s output data are collected and saved into files accordingly with a sampling rate of 100 Hz. The angular rate collected included: ±5 °/s, ±10 °/s, ±15 °/s, ±20 °/s, ±40 °/s, ±60 °/s, ±100 °/s and ±150 °/s.

### 2.3. Variance Calculation

After data collection, the different angular rate gyroscope Z axis output data files are loaded to calculate the data variance. The different angular rate measurement data variances are shown in Table 1.

Table 1 indicates that the gyroscope output data variance increased with the increase of ω’s absolute values. Moreover, there was an ascertainment relation. According to the gyroscope input angular rate and variance data, the mapping relation between the angular rate and the gyroscope output data variance could be fitted. On the basis of the mapping relation, the output data variance can be predicted with the known angular rates.

### 2.4. Dynamic Variance Model

The proposed approach utilized the high-order function fitting method to fit the mapping function between variance and angular rate, to get the 4-order dynamic variance model as follows:

(1) $$V_{\omega }=0.0003\omega^{4}+0.0005\omega^{2}-0.0003\omega +0.0267$$

**Figure 1:**
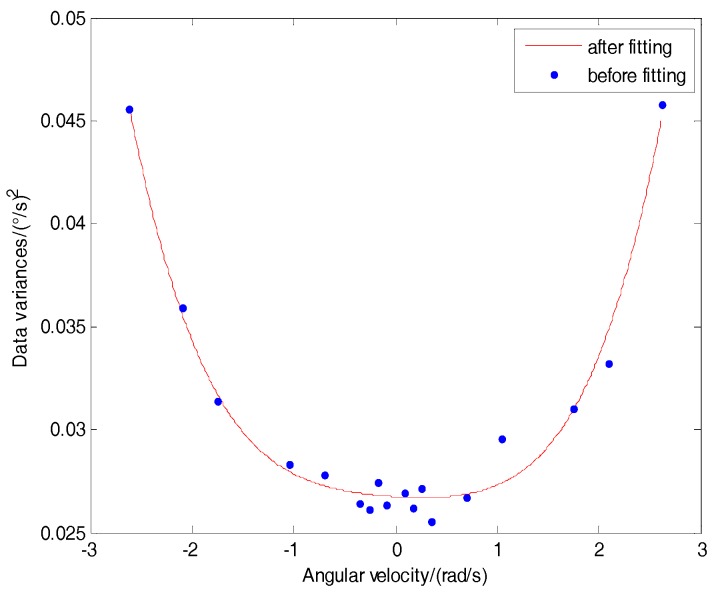
Raw data variance fitting.

The fitting curve between the gyroscope input angular rates and data variances is shown in Figure 1.

From Figure 1, it is shown that the fitting curve can describe the correlation between the gyroscope output data variance and the angular rate. In practical systems, according to Equation (1) and the real-time measuring angular rate, the random signal variance $V_{\omega }$ can be predicted.

In Kalman filtering, which takes data variances as parameters, the proposed approach adjusts the Kalman filtering measuring noise coefficients based on the angular rate-related data variance. The proposed approach eliminates the influence of angular rate-related output data variance on filtering efficiency. 

## 3. Adaptive Filtering Method Based on Online Measuring Noise Coefficient Adjustment

The random signal modeling and filtering method is an effective method to compensate for the MEMS gyroscope’s dynamic random error [14]. Generally, time sequence modeling methods are utilized to build a MEMS gyroscope’s random error model [17], which when combined with Kalman Filtering realizes random error modeling compensation [18].

In previous work, the gyroscope static output data variance was set as Kalman Filtering measuring noise coefficients [18]. Therefore, when the gyroscope output data variance changes with angular rate, the proposed method adjusts measuring of the noise coefficients online according to angular rate-related changing data variance. The Kalman Filtering measuring noise coefficients online adjustment based on the dynamic variance model improves filtering performance, realizing adaptive filtering.

### 3.1. Non-Stationary Random Signal Modeling Methods

ARMA model and ARIMA model separately are efficient methods to describe stationary data and no-stationary data [16]. Because the MEMS gyroscope’s output random signal has a weak linear trend item, the output random signal is a non-stationary time sequence, which needs to be modeled with the ARMIA model [26]. In engineering applications, common methods have applied the Difference method to deal with the MEMS gyroscope output data, obtaining stationary signals [27]. The ARMA model is built with the stationary data. After that, the methods utilize the difference operator to transfer in a reverse manner from the ARMA model to the non-stationary data corresponding ARIMA model. The ARIMA model shows original non-stationary time sequence statistic characteristics [26].

Given $\nabla^{d}$ as a first difference operator, *B* as a backward shift operator [16], the first order difference to the non-stationary time sequence $\left\{x_{k}\right\}$ can be expressed as

(2) $$\nabla x_{k}=x_{k}-x_{k-1}=(1-B)x_{k}\;$$

In Equation (2), $Bx_{k}$ stands for the last moment data $x_{k-1}$. The signal difference procedure continues until the data show as stationary characteristics. Commonly, the data satisfy stationary requirements after one or two difference calculations [27]. After the dth-order difference, a new stationary time sequence$\left\{z_{k}=\nabla^{d}x_{k}\right\}$ is formed, where $\nabla^{d}$ is set as the dth-order difference operator. For $\left\{z_{k}\right\}$, the ARMA (*n*,*m*) model is built as:

(3) $$z_{k}=\sum_{i=1}^{n}\varphi_{i}z_{k-i}-\sum_{j=1}^{m}\theta_{j}a_{k-j}+a_{k},a_{k}\sim NID(0,\sigma_{a}^{2})$$

where $\varphi_{i}$ and $\theta_{j}$ are coefficients that satisfy stationary and invertible conditions, respectively [16], $a_{k}$ is white noise which is an uncorrelated random variable with mean zero and constant variance $\sigma_{a}^{2}$. Replacing $\left\{z_{k}\right\}$ with $\left\{\nabla^{d}x_{k}\right\}$, the original data non-stationary time sequence model ARIMA (*n,d,m*) can be written as:

(4) $$\Phi (B)\nabla^{d}x_{k}=\Theta (B)a_{k}$$

where $\nabla^{d}={\left(1-B\right)}^{d}$, the term $\Phi (B)=1-\varphi_{1}B-\cdots -\varphi_{n+d}B^{n+d}$ is an autoregressive coefficient polynomial of the model, and $\Theta (B)=1-\theta_{1}B-\cdots -\theta_{m}B^{m}$ is a moving smoothness coefficient polynomial. Operator B stands for the backward shift operator.

Using the method above mentioned in [28], a non-stationary time sequence model which can describe the random signal’s changing regular pattern of the MEMS gyroscope is built.

### 3.2. Kalman Filter Design with ARIMA Model-Based State Equation

The MEMS gyroscope’s random error is the system noise-driving output. It can be seen as a non-stationary time sequence, and can be described by the ARIMA model in Equation (4) [28]. The ARIMA model reveals the MEMS gyroscope’s current output data and previous output data relations. The relation written in form of a state-space representation can be the Kalman Filter’s state equation [28]. Section 3.2 mainly discusses the previous filtering method in [28] and its limitation. Based on this method, the proposed method ameliorated its performance under dynamic conditions.

The Kalman filtering system’s state equation and measuring equation can be written as:

(5) $${\{\begin{array}{l} X_{k}\;=\;\Phi X_{k-1}\;+\;GW_{k}\\ Z_{k}\;=\;HX_{k-1}\;+\;V_{k} \end{array}}_{}^{}\;$$

where $\Phi \;=\;\left[\begin{array}{cc} \Phi_{1},\Phi_{2},\cdots ,\Phi_{n+d-1} & \Phi_{n+d}\\ I_{n+d-1} & 0 \end{array}\right]$ is the system state transition matrix, $G\;=\;\left[\begin{array}{cc} G_{1},G_{2},\cdots ,G_{m-1} & G_{m}\\ I_{m-1} & 0 \end{array}\right]$ is the system noise driven matrix, $W_{k}$ is the system noise, H is the system measuring matrix, and $V_{k}$ is the measuring noise.

The system state vector $X_{k}$ is determined according to the ARIMA (*n*, *d*, *m*) model’s autoregressive coefficient polynomial *n* and difference order *d*, represented by $X_{k}={\left[\begin{array}{cccc} x_{k} & x_{k-1} & \cdots & x_{k-\left(n+d-1\right)} \end{array}\right]}^{T}$. In the system state transition matrix $\left\{\Phi_{1},\Phi_{2},\cdots ,\Phi_{n+d}\right\}\;=\;\left\{\varphi_{1},\varphi_{2}\cdots \varphi_{n+d}\right\}$; in the system noise driven matrix G, coefficients $\left\{G_{1},G_{2},\cdots ,G_{m}\right\}$ take the values of $\left\{\theta_{1},\theta_{2},\cdots ,\theta_{m}\right\}$. The MEMS gyroscope’s real-time output data is taken as the system measuring values, and $H\;=\;\left[\begin{array}{cc} 1 & 0_{n+d-1} \end{array}\right]$. The measuring noise $V_{k}$ is random data variance. It is a fact that the value accuracy of $V_{k}$ influences the filtering performance. For the immobilized statistic characteristics’ random data, previous methods calculated $V_{k}$ according to the prior data; for volatile statistic characteristics random data filtering, the value adjustment of $V_{k}$ according to real-time gyroscope output data variance should be conducted. In Section 3.3, online adjustment based on the dynamic variance model is discussed.

### 3.3. Measuring Noise Coefficient Online Adjustment-Based Adaptive Filtering Approach

The improvement work achieved in this paper was based on the previous work in Section 3.1 and Section 3.2. The dynamic random error model was built in Section 2, and was utilized to adjust the measuring noise coefficients online in Section 3.3.

In Equation (5), data sequence $\left\{V_{k}\right\}$ is the noise sequence, which reveals measuring signal random characteristics. Its variance can be shown as:

(6) $$E\left[V_{k}V_{j}^{T}\right]=R_{k}\delta_{kj}\;$$

where $R_{k}$ is the Kalman filter measuring noise coefficient [27,28], to determine weighting coefficients between the measuring variable and system estimation variable. The accuracy of $R_{k}$ influences the filtering results.

The proposed method takes the current moment output data as measuring values, and the gyroscope’s output data variance as $R_{k}$. Due to the fact that the gyroscope output data variance changes with input angular velocity, $R_{k}$ can be described by the function of angular velocity as:

(7) $$R_{k}\left(\omega \right)=f\left(\omega \right)$$

where f(ω) is determined by the gyroscope’s dynamic random characteristics. With different gyroscopes, function expressions are not the same, and need to be calibrated before use. Therefore, Section 2 of this paper determines the ADS16488 gyroscope’s output data variance with the input angular velocity’s non-linear mapping function through experimental testing and data fitting methods, shown as Equation (1). Thus we can get:

(8) $$R_{k}\left(\omega \right)=V_{\omega }=0.0003\omega^{4}+\;0.0005\omega^{2}-0.0003\omega +0.0267$$

With Equation (8), according to real-time measured angular velocity ω, utilizing online calculated R(ω)k to substitute the Kalman filter $R_{k}$ one can achieve online adaptive adjustment of the Kalman filter’s measuring noise coefficients.

## 4. Experiments and Results

### 4.1. Experimental Equipment

**Figure 2:**
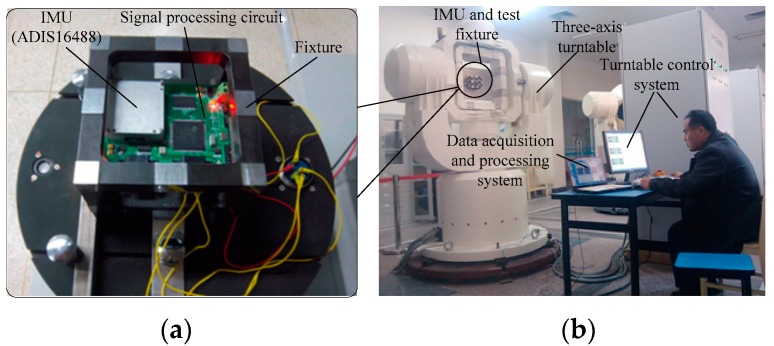
Experimental system. (**a**) Inertial measurement unit (IMU) and fixture. (**b**) Integral structure.

The experimental equipment including the high accuracy three-axis angular rate turntable, data collection and processing system, the inertial measurement unit (IMU) ADIS16488, and the hexahedron mounting fixture, are separately shown in Figure 2a,b.

In Figure 2a, ADIS16488 is a MEMS inertial measurement unit (IMU) and includes a three-axis gyroscope and a three-axis accelerometer. This paper mainly studies the axis Z gyroscope dynamic error characteristics and the random error compensation method. The gyroscope’s measuring range is ±450 °/s, and its zero-bias stability is 5.1 °/h, and bias repeatability at −40 °C to 80 °C is ±0.2 °/s. The mounting fixture is an integrated machining hexahedron, with setting of the surface’s parallelism and vertical accuracy of 10 arc-seconds. The ADIS16488 signal pre-treatment circuit is a DSP28335 based signal transition circuit, which reads the ADIS16488 Z axis gyroscope signal output, and sends it through a serial port after pre-processing. The three-axis turntable’s three-axis angular rate ranges are: outer frame shaft 150 °/s, middle frame shaft 200 °/s, inner frame shaft 400 °/s. The turntable position accuracy is 1 arc-second, and under 100 °/s its relative rate accuracy is $10^{-5}$. A high-accuracy turntable was installed firmly on the customized groundsill, which as well as high accuracy, had good stabilization and did not bring additional errors to the gyroscopes. In the experiment, ADIS16488 was fixed onto the inner cast of the turntable, positioning the axis-Z gyroscope’s sensitive shaft vertically upwards. Then the axis-Z gyroscope output data were collected to verify the proposed method in this paper.

### 4.2. Experimental Procedure

**Figure 3:**
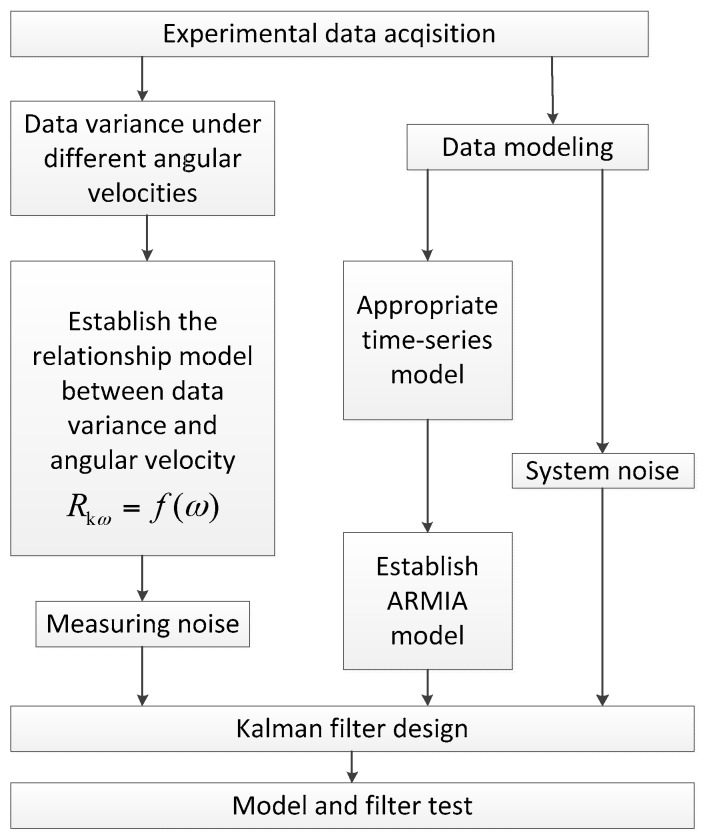
Experimental scheme.

On the basis of theoretical analysis, the experimental instruments and equipment in the laboratory were utilized to verify the studied dynamic random error compensation method. The experimental scheme is in Figure 3.

First of all, on the high accuracy three-axis rate turntable, different angular rate ADIS16488 axis-Z gyroscope output data were collected separately. Then the random error statistic characteristics of the different angular rates data were analyzed, building the relevant ARIMA model and dynamic variance model (in Section 2). After that, with respect to the ARIMA model, the Kalman filter was designed. According to the dynamic relationship model of data variance and angular rate, the proposed method adjusted the Kalman filter measuring noise coefficients online. Finally, the constant angular rate and continuous changing angular rate gyroscope output data were utilized separately to verify the built model and filter.

### 4.3. Verification Experiments

#### 4.3.1. Kalman Filtering State Equation Utilizing the Time Sequence Model

Under the different angular velocities, the gyroscope output data were all non-stationary data. Through first-order difference, stationary data $\left\{z_{k}\right\}$ was acquired. Through the nine groups of experimental data, the comprehensive analysis, AR(*3*,*0*) model was determined to reveal the statistic characteristics of the stationary data $\left\{z_{k}\right\}$. Parameters in AR(*3*,*0*) were as follow:

$$z_{k}=0.242z_{k-1}+0.220z_{k-2}-0.462z_{k-3}+a_{k}\;$$

Substitute $z_{k}=x_{k}-x_{k-1}$ in Equation (9), the original data’s ARMIA(*3*,*1*,*0*) model is determined as follow:

(10) $$x_{k}=1.242x_{k-1}-0.023x_{k-2}-0.682x_{k-3}+0.462x_{k-4}+a_{k}\;$$

**Figure 4:**
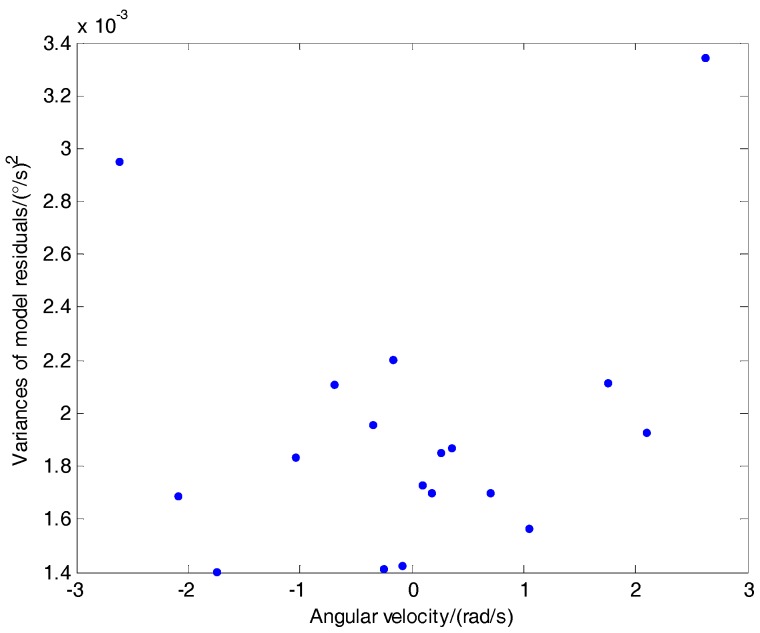
Remaining random noise variance.

The verification experiment applied the ARMIA(*3*,*1*,*0*) model to different angular rate output data, acquiring the remaining random noise data $\left\{a_{k}\right\}$ after modeling as in Figure 4:

As Figure 4 depicts, the different angular velocity’s left random noise variance is between 0.0014 ${\left({}^{\circ}/s\right)}^{2}$ and 0.0034 ${\left({}^{\circ}/s\right)}^{2}$, which is smaller and random without obvious tendency compared with the raw data variance in Table 1. Thus the constructed ARMIA(*3*,*1*,*0*) model is able to reveal different angular rates of the gyroscope’s output data random statistic characteristics. Moreover, the proposed method took the ARMIA(*3*,*1*,*0*) model as the Kalman Filtering state model to acquire the Kalman Filtering state equation.

#### 4.3.2. Kalman Filter Design

Equation (10) revealed the gyroscope’s several adjacent moments output data connection, describing the dynamic random data changing regular pattern. According to Equation (10), the Kalman filter state variables were chosen as $X_{k}\;=\;{\left[\begin{array}{cccc} x_{k} & x_{k-1} & x_{k-2} & x_{k-3} \end{array}\right]}^{T}$, where $x_{i}$ is the time point *i* gyroscope output data. From Equation (10), the correspondent system state transition matrix Φ in Equation (5) is:

$$\Phi =\left[\begin{array}{cccc} 1.242 & -0.023 & -0.682 & 0.462\\ 1 & 0 & 0 & 0\\ 0 & 1 & 0 & 0\\ 0 & 0 & 1 & 0 \end{array}\right]\;$$

The system noise matrix *G* is shown as:

$$G=\left[\begin{array}{cccc} 1 & 0 & 0 & 0\\ 0 & 0 & 0 & 0\\ 0 & 0 & 0 & 0\\ 0 & 0 & 0 & 0 \end{array}\right]\;$$

The current moment gyroscope output data is chosen as the measuring value, the system observing matrix H is: $H=\left[\begin{array}{cccc} 1 & 0 & 0 & 0 \end{array}\right]$.

Due to the fact that the model residuals fluctuate with variation of angular rates, the proposed approach chose 0.0019, which is the mean value of the left variance of the ARMIA model under the different angular rate in Figure 4, to be the system noise, and used $R_{k\omega }$ calculated in real-time by Equation (8) to adjust the measuring noise coefficients in the Kalman filtering. Therefore, adaptive adjustment of measuring noise coefficients under different angular velocity was processed.

#### 4.3.3. Different Angular Rate Experiment Verification

The verification experiment utilized experimental data to confirm the proposed approach. Expediently, the proposed method was marked as Kalman Filtering with adjustments (A-KF method). The compensation method without the measuring noise online adjustment mentioned in reference [14,17,18] was marked as the Kalman Filtering method based on the ARMIA model (KF method). Comparison between the KF method and the A-KF method was discussed and tested to verify the effectiveness of the proposed approach.

(1) Constant Rotation Rate Filtering Experiments

**Figure 5:**
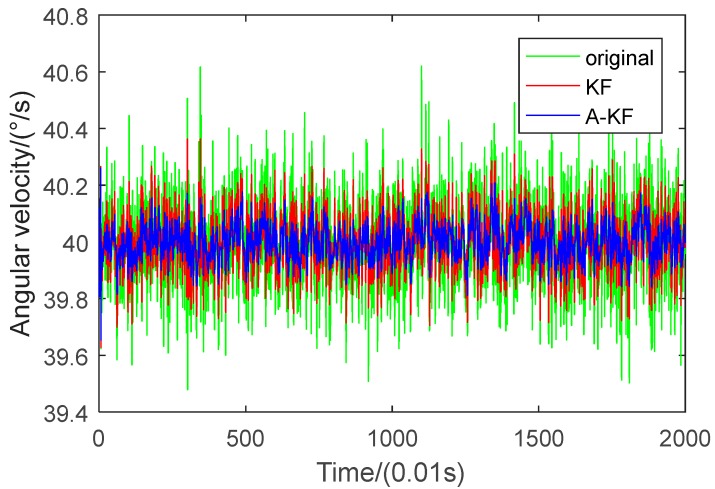
Comparison of filtering results.

Rotation rate of 40 °/s gyroscope output data were collected. Raw data separately applying to the A-KF method and the KF method are shown in Figure 5.

Figure 5 indicates that the random error restriction effectiveness with the A-KF method is better than with the KF method. The statistics before and after filtering are shown in Table 2.

In Table 2, variances after filtering were separately processed with the proposed approach (A-KF) and the KF method, which did not adjust measuring the noise online; the percentages are the ratios of the filtering data variance and original data variance. 

Under an angular velocity of 40 °/s, the variance of the original data is 0.0267. Using KF, the data variance of 0.0084 is 31.5% of the original data; Using A-KF, the variance of the original data reduces to 0.0040, which is 15.0% of the original data, and 47.6% of the KF without online adjustment.

Data analysis results show that after using the proposed approach to deal with data, random errors are restricted more efficiently.

(2) Continuous-Changing Angular Rate Filtering Experiments

**Figure 6:**
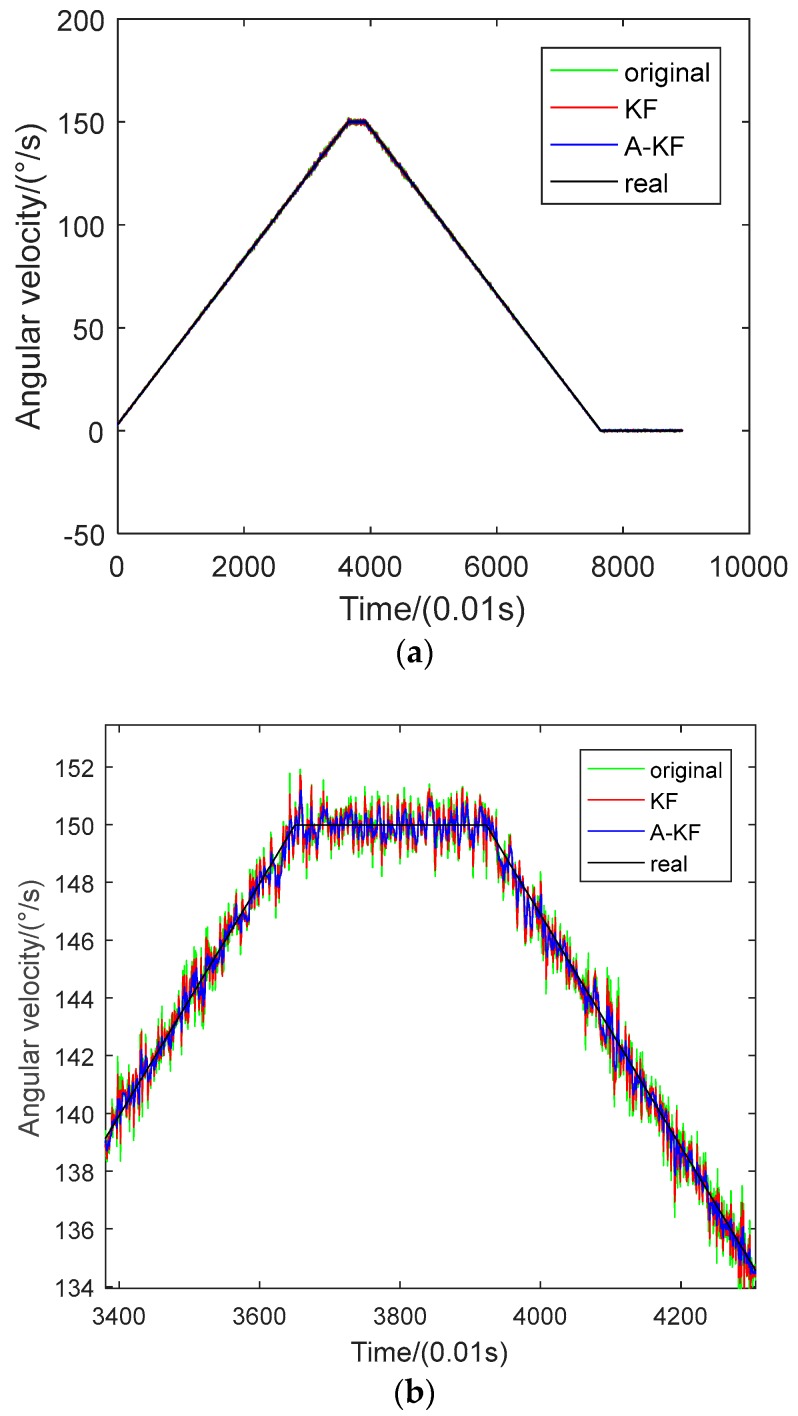
Data comparison between filtering with the Kalman filtering method based on the ARMIA model (KF) method and the Kalman filtering with adjustments (A-KF) method. (**a**) Entirety data comparison; (**b**) part of the data zoomed in.

**Figure 7:**
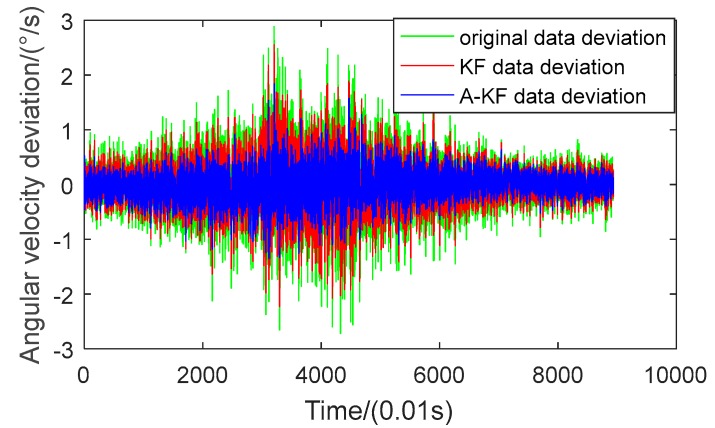
Angular rate error comparisons between the KF method and the A-KF method.

To verify the feasibility of the proposed method, continuous-changing angular rate dynamic filtering experiments were arranged. In the dynamic filtering experiment, data were collected correspondent to the angular rate: first from 3 °/s to 150 °/s with a constant angular acceleration, then retained for 3 s and rotated with a decreasing angular velocity, returned to the static state and maintained for 12 s. During the rate-changing period, the axis-Z gyroscope ADIS16488 output data were collected and saved into files. Results before and after filtering are shown in Figure 6 and Figure 7. Figure 6 compares data separately utilizing the KF method and A-KF method; Figure 7 shows the angular velocity errors filtered separately with the KF method and A-KF method. 

In Figure 6, after both the filtering methods, the data can track the turntable rates. Moreover, the angular rate error with the proposed approach is smaller than with the KF method without real-time measuring noise adjustment. From Figure 7, the original data random error ascends with the enlargement of the angular rate, and descends with the decrease of the angular rate. During the whole method the process variance of the original data is 0.2686. Using the KF method without real-time measuring noise adjustment, the data variance is 0.0849, 31.6% of the original data. Using the proposed approach to filtering, the variance of the original data is 0.0526, 19.6% of the original data, and 62.0% of the KF method. Therefore, from the data comparison, the proposed filtering approach could achieve filtering effectiveness under continuous changing angular rate.

(3) Long Term Constant Rotation Rate Filtering Experiments

**Figure 8:**
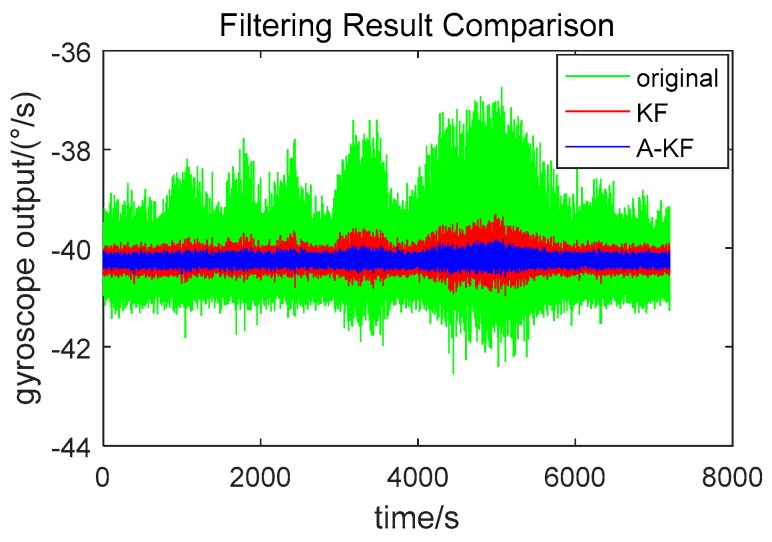
Long term data comparison between the proposed method and KF method.

**Figure 9:**
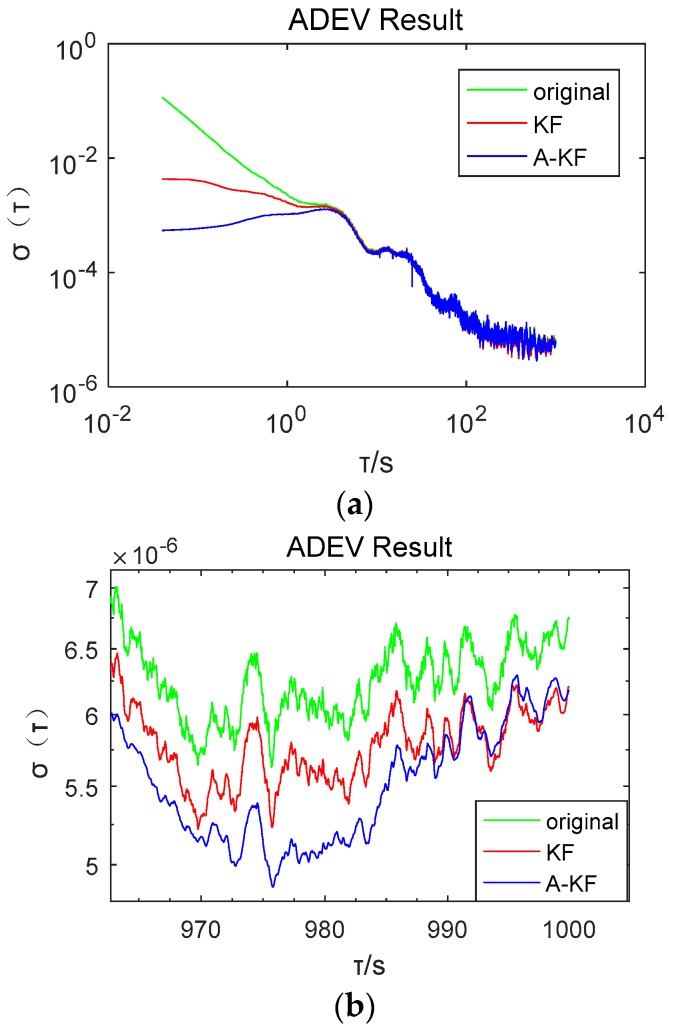
Long term Allan Deviation (ADEV) double logarithmic chart. (**a**) Entirety ADEV double logarithmic chart; (**b**) part of the long term ADEV chart zoomed in.

To verify the long term stability of the proposed method, rotation rate of 40 °/s gyroscope output data were collected within 2 h. Raw data separately applied to the proposed A-KF method and the KF method with common Kalman filtering without measuring noise coefficient adjustment are shown in Figure 8. Moreover, the Allan Deviation (ADEV) was calculated and ADEV double logarithmic chart is shown in Figure 9.

Figure 8 indicates that random error restriction effectiveness with the A-KF method is better than with the KF method in the long term. Under an angular velocity of 40 °/s, the variance of the original data is 0.0985. Using KF, the data variance of 0.0104, is 10.6% of the original data; using the proposed A-KF method, the variance of the original data reduces to 0.0035, which is 33.7% of the KF without online adjustment. In Figure 9, it is shown that in the period of T (the ADEV integration time [29]) equal to 0.01–1 s, the ADEV falls to 12.61% of the KF method after utilizing the proposed A-KF method. In the period of T equal to 100–1000 s, the ADEV shows a decrease to 98.76% of the KF method after utilizing the A-KF method. Data analysis results show that the proposed approach is effective in dealing with long time data random errors.

## 5. Conclusions

In conclusion, the proposed approach analyses the relations between the gyroscope random error and the angular rate, by building a fourth-order dynamic variance model of the gyroscope output data and angular rate. After that, the proposed method of online-adjustment of the Kalman Filtering measuring error coefficients based on the dynamic variance model, realizes MEMS gyroscope output signal random error adaptive filtering. Moreover, the proposed method was verified through a constant angular rate and continuous-changing angular rate turntable experiment. The proposed approach could be applied to compensate for MEMS gyroscope dynamic random error and obtain an estimation of the MEMS gyroscope dynamic output signal.

## Figures and Tables

**Table 1:** Different angular rate gyroscope output data.

ω (°/s)	Data Variance	ω (°/s)	Data Variance
−5	0.0264	5	0.0269
−10	0.0275	10	0.0262
−15	0.0261	15	0.0272
−20	0.0264	20	0.0256
−40	0.0278	40	0.0267
−60	0.0283	60	0.0296
−100	0.0314	100	0.0310
−120	0.0359	120	0.0332
−150	0.0456	150	0.0458

**Table 2:** The filtering result comparison of the Kalman filtering with adjustments (A-KF) method and the Kalman filtering method based on the ARMIA model (KF) method.

Angular Velocity (°/s)		40
Variance of original data		0.0267
KF	Variance after filtering	0.0084
	Percentage	31.5%
A-KF	Variance after filtering	0.0040
	Percentage	15%
